# PD1 is expressed on exhausted T cells as well as virus specific memory CD8+ T cells in the bone marrow of myeloma patients

**DOI:** 10.18632/oncotarget.25882

**Published:** 2018-08-10

**Authors:** Anne-Marit Sponaas, Rui Yang, Even Holth Rustad, Therese Standal, Aud Solvang Thoresen, Camilla Dao Vo, Anders Waage, Tobias S. Slørdahl, Magne Børset, Anders Sundan

**Affiliations:** ^1^ Department of Clinical and Molecular Medicine, Myeloma Research Center, Norwegian University of Science and Technology, Trondheim, Norway; ^2^ Centre of Molecular Inflammation Research, Centre of Molecular Immune Regulation, Norwegian University of Science and Technology, Trondheim, Norway; ^3^ Department of Hematology, St. Olavs University Hospital, Trondheim, Norway; ^4^ Department of Immunology and Transfusion Medicine, St. Olavs University Hospital, Trondheim, Norway; ^5^ Department of Medicine, Gjøvik Hospital, Gjøvik, Norway

**Keywords:** myeloma, bone marrow, checkpoint molecules, CD8 T cells, exhaustion

## Abstract

Characterization of CD8+ T cells in the tumor microenvironment (TME) is important to predict responses to checkpoint therapy. The TME in multiple myeloma is the bone marrow, which also is an immune organ where immune responses are generated and memory cells stored. The presence of T cells with other specificities than the tumor in the bone marrow may affect the search for biomarkers to predict responses to immunotherapy in myeloma. Here, we found similar proportions of PD1+ CD8+ T cells and similar levels of PD1 expression on CD8+ T cells in the bone marrow of myeloma patients and healthy controls. PD1 expression on CD8+ T cells did not correlate with tumor load suggesting that at least some of the PD1+ CD8+ T cells were specific for non-myeloma antigens. Indeed, PD1+ EBV-specific CD8+ T cells were detected it the bone marrow of patients. Terminal effectors (Teff), effector memory (Tem) and central memory (Tcm) cells as well as exhausted T cells were all found in the myeloma bone marrow. However, myeloma patients had more terminal effectors and fewer memory cells than healthy controls suggesting that the tumor generate an immune response against myeloma cells in the bone marrow. The presence of CD8 EOMES^high^ Tbet^low^ T cells with intermediate levels of PD1 in myeloma patients suggests that T cell types, that are known to be responsive to checkpoint therapy, are found at the tumor site.

## INTRODUCTION

Anti-PD1 treatment has been effective in clinical trials of several advanced hematological and solid cancers [1, 2]. Primary myeloma cells as well as dendritic cells from patients express the ligand PDL1 (CD274) [3–6]. In addition, proportions of CD8+ and CD4+ T cells as well as NK cells in the bone marrow of myeloma patients express PD1 (CD279) [3, 7]. This led to the initiation of several clinical trials with anti-PD1 antibodies in myeloma. Although many of these clinical trials are not published yet, mixed responses to PD1 treatment have been reported so far [8]. Recently it was reported that two phase III clinical trials combining standard treatment of dexamethasone and lenolidamide or pomalidomide with the anti-PD1 inhibitor pembrolizumab (Keytruda) were terminated due to more deaths in the pembrolizumab arm (Keynote 183, Keynote 185, www.FDA.gov).

It is now becoming clear that immunogenic tumors respond best to checkpoint therapy, as they may have a larger repertoire of tumor-reactive T-cell clones [9, 10]. PD1 is upregulated on T cells after activation and this represents a natural regulation of potentially dangerous immune responses [11]. The rationale behind anti-PD1 treatment is to abrogate the exhausted state of the patient's tumor specific CD8+ T and NK cell responses. Although this appears to be the case in animal studies and recently reported to be the case in clinical studies [12, 13], it is not entirely clear what happens in patients treated with checkpoint inhibitors. PD1 expression on T cells at the tumor site has been proposed to be a prerequisite for successful treatment, as lack of PD1 expression on tumor infiltrating T cells (TILs) were associated with reduced response to checkpoint therapy [11]. These results came from studying solid tumors where one could assume that the infiltrating T cells would be responsive to the tumor itself. Multiple myeloma resides in the bone marrow, an immunological organ where T cells are activated by APCs, and it is also a site for storage for T memory cells [14]. Although PD1-treatment has been successful in an animal model on myeloma [3] and anti-PDL1 antibodies have reinvigorated exhausted T cells from a myeloma patient to kill myeloma cells *in vitro* [4], it is not clear whether anti-PD1/PDL1 treatment induce anti-tumor activity by reinvigorating myeloma-specific exhausted T cells in myeloma patients. PD1 is not only expressed on dysfunctional T cells, such as anergic and exhausted T cells, but also on terminal effector T cells and memory T cells [15]. Thus, in order to understand how PD1/PDL1 therapy would function in multiple myeloma, it is important to characterize effector functions and the phenotypes as well as the specificity of the CD8+ T cells in the myeloma TME. In this study we tested whether PD1 expression on CD8+ T cells from bone marrow correlated with tumor load and investigated whether these T cells could respond to autologous myeloma cells *in vitro*. We found that a large proportion of the bone marrow PD1+ CD8+ T cells were T effector or T memory cells. However, these PD1+ CD8+ T cells failed to degranulate in the presence of autologous myeloma cells and PD1 antibody, suggesting specificity to non- tumor antigens. This was supported by the presence of PD1+, EBV-specific T cells in the bone marrow of patients.

## RESULTS

### Tumor load does not correlate with the proportion of PD1+CD8+ T cells in the bone marrow or the level of PD1 expression

Tumor load is an important factor for an efficient checkpoint therapy [12]. If one assumes that antigens from myeloma cells are responsible for activation of T cells and the subsequent upregulation of PD1 in tumor-reactive CD8+ T cells, there should be either more PD1+ cells or higher levels of PD1 on the CD8+ T cells from myeloma patients compared to healthy controls. We set out to determine PD1 expression on CD8+ T cells in bone marrow aspirate from a cohort of myeloma patients and healthy controls enrolled in the Norwegian Myeloma Biobank Study/Biobank1 (The clinical information of the patients is shown in Supplementary Table 1 and the gating strategy in Supplementary Figure 1A). Fresh bone marrow from patients and controls was used assuming that this would give a more relevant picture of the bone marrow status than using thawed cells.

**Figure 1:**
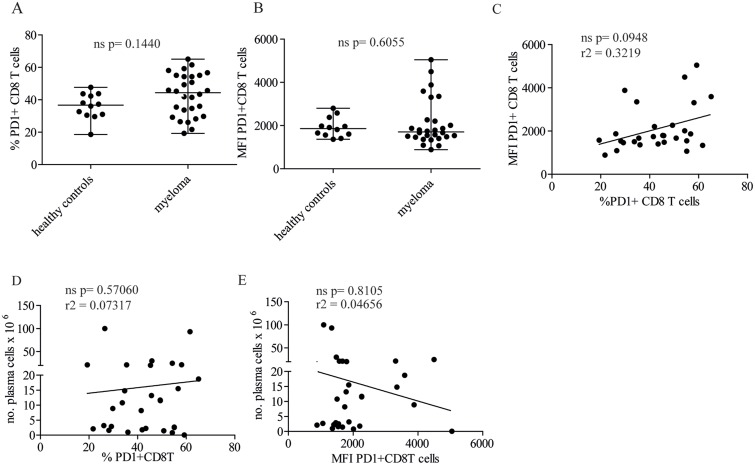
Neither proportion of PD1+ CD8+ T cells in the bone marrow nor levels of PD1 expression correlate with tumor load **(A)** Percent PD1+ cells of the CD8+ population and **(B)** MFI (Median Fluorescence Intensity) of PD1 on CD8+ T cells from crude bone marrow from healthy controls (n=12) and myeloma patients (n=28). Patient data is shown in Supplementary Table 1. Bone marrow cells were stained with antibodies against CD3, CD8, CD4 and CD279 (PD1). The gating strategy is shown in Supplementary Figure 1A. Briefly, gates were set on CD3 and CD8 and % PD1+ cells were determined using FMO control. Figure shows median values with range. Each dot represents one patient. P values were calculated from a Mann–Whitney *U* test. **(C)** Plots show relationship between the percentage of PD1+CD8+ T cells and the MFI of PD1 on bone marrow cells from myeloma patients shown in A/B **(D, E)**. Plot shows relationship between number of CD138+ plasma cells obtained from 20 ml bone marrow aspirate and percent PD1+ CD8+ T cells (D) MFI of PD1 on CD8+ T cells (E) of patients in figures A/B. Each dot represents one patient.

There were no significant differences in the percent of cells with PD1 (Figure 1A) or in the levels of PD1 (Figure 1B) expressed on CD8+ bone marrow T cells between healthy controls and myeloma patients. Around 40% of the CD8+ T cells in the bone marrow of myeloma patients (median 44.4) and healthy controls (median 36.7) expressed PD1. Myeloma patients showed a greater individual variation than controls in proportion of PD1+ CD8+ T cells ranging from around 70% to less than 20% PD1+ cells (Figure 1A). The majority of the patients, (22 out of 28) had MFIs clustered around the median (Figure 1B). A small number of patients expressed high PD1 levels, 6 out of 28 had CD8+ T cells with MFIs that separated clearly from the main cluster (Figure 1B). Two of these patients with high PD1 expression had relapsed after treatment and four were not previously treated (see Supplementary Table 1 patients marked^*^). Interestingly, there was no significant correlation between the expression levels and proportions of PD1+ CD8+ T cells (Figure 1C). TCR ligation will up-regulate PD1 expression on T cells [16], and high expression levels of PD1 is, at least in viraemic patients, an indication of the extent of virus-specific T cell activation followed by exhaustion [17]. If the induction of PD1 is a result of CD8+ T cell activation by the tumor cells, then one could predict that tumor load would correlate with expression levels of PD1 or the proportion of PD1+ CD8+ T cells. However, there was no correlation between the percent PD1+ CD8+ T cells and the number of plasma cells (PC) isolated from the bone marrow aspirates (Figure 1D), or the bone marrow PC cellularity (% bone marrow PC) estimated from bone marrow smears (Supplementary Figure 1B). Likewise, expression levels of PD1 did not correlate with the number of isolated PC (Figure 1E), or % PC from bone marrow smears (Supplementary Figure 1C). The number of plasma cells isolated from bone marrow correlated with % PC in bone marrow smears (Supplementary Figure 1D, Thyness and Waage unpublished data). We are aware that % PC and plasma cell yield from aspirates may not always reflect the total tumor burden or disease, but we argue that it can be used as a measure of immune cells and their activation at the aspiration site.

Other indicators of tumor load such as level of M component did not correlate with percent PD1+ cells or level of PD1 on the CD8+ T cells (data not shown). The patients with high ISS score (III) did not have higher levels of PD1 or more PD1+ CD8+ T cells than the ones with lower scores (data not shown). The majority of the patients did not have elevated CRP values or clinical signs of infection (Supplementary Table 1 and data not shown).

### Majority of PD1+ CD8+ T cells are Granzyme B+, IFNγ− and TNFα-producing cells

**Figure 2:**
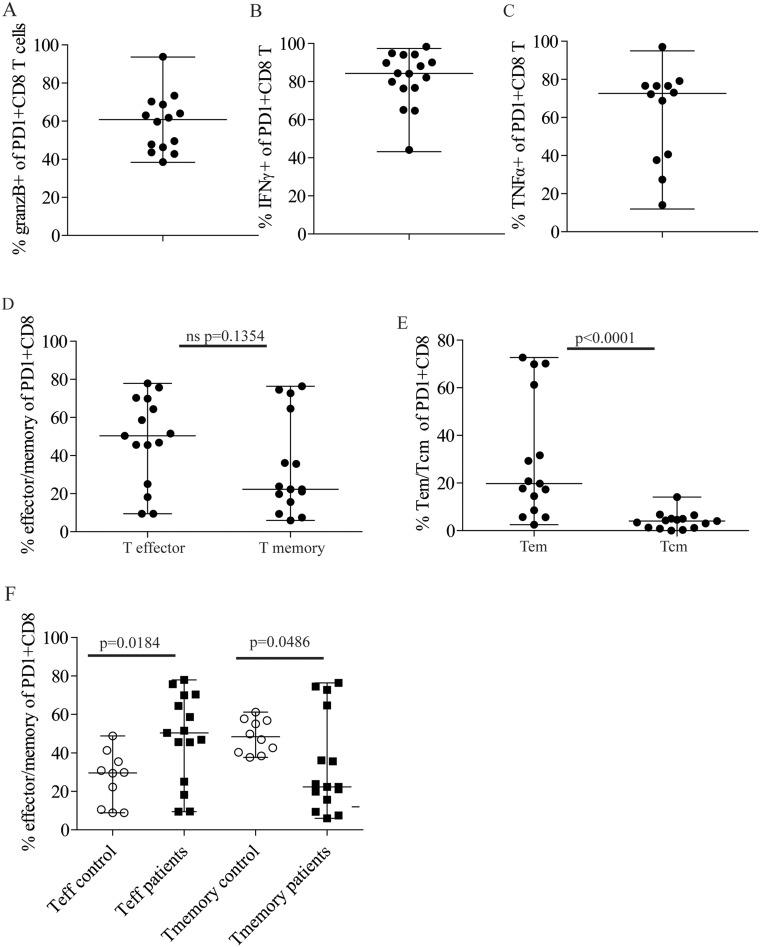
Majority of PD1+CD8+ T cells in patient bone marrow are Granzyme B+ and produce IFNγ and TNFα **(A)** Percent Granzyme B+ cells of PD1+ CD8+ T cells in the bone marrow of myeloma patients (n=14). Crude bone marrow was surface stained with anti CD3, CD4, CD8 and PD1 followed by intracellular staining with anti Granzyme B or isotype control. Gating strategy is shown in Supplementary Figures 1A and 2A. Figure shows median value and range. **(B, C)** Percent PD1+CD8+ T cells producing cytokines in the bone marrow of patients. BMMCs were stimulated as described in Materials and Methods. The cells were then surface stained with anti CD3, CD4, CD8 and PD1 followed by intracellular staining with anti IFNγ (n=16) (B) TNFα (n=12) (C) or isotype control. Gates were set as shown in Supplementary Figure 2C, D. **(D)** Frequency of T effector (Teff) (CD45RA+CD45RO-CCR7-) and T memory (combined Tem and Tcm) (CD45RO+CD45RA-CCR7+ and CCR7-) of the PD1+ CD8+ T cells (n=15). **(E)** Frequency of Tem (CD45RO+CD45RA-CCR7-) and Tcm (CD45RO+CD45RA-CCR7+) of PD1+CD8+ T cells of patients in D. **(F)** Frequency of Teff and T memory (Tem and Tcm) of PD1+CD8+ T cells in healthy controls (open circles, n= 10) and patients (closed squares, n=15). Gating strategy is shown in Supplementary Figure 3A. Figures shows median value and range. P values were calculated with a Mann–Whitney *U* test.

We next characterized the PD1+ CD8+ T cells in the bone marrow phenotypically and functionally. Most of the PD1+ CD8+ T cells in the bone marrow were Granzyme B+ cytototoxic T cells and they were present in all patients and in healthy controls (Figure 2A, Supplementary Figure 2B). The proportion of Granzyme B+ cells within the PD1+ population varied somewhat in the patients from around 40 to 100% (Figure 2A), but as both patients and healthy controls had similar percentages of PD1+ cytotoxic T cells (Supplementary Figure 2B), this variation may not be related to the disease. The functional activity of the PD1+ CD8+ populations, however, may differ in controls and patients. For example, the proportion of effectors, memory and exhausted cells could vary. In addition, the antigen-specificity could also be different, as one would not expect to find myeloma antigen specific T cells in healthy controls. Cytokine-producing terminal effectors and memory cells, as well as exhausted CD8+ T cells all express PD1, and the bone marrow is a site of memory cells specific to various pathogens [14]. Therefore, some of the PD1+ CD8+ T cells could be memory cells that recognize antigens other than myeloma antigens. Indeed, all patients had PD1+ CD8+ T cells that produced IFNγ and TNFα (Figure 2B, C, Supplementary Figure 2C, D) in their bone marrow. All patients had > 40% of their PD1+ CD8+ T cells producing IFNγ (Figure 2B), and 9/10 had >20% PD1+ TNFα producers (Figure 2C). The proportion of the cytokine producing PD1+ CD8+ T cells varied among the myeloma patients. This variation could not be attributed to tumor load, any clinical parameters, or even levels or proportion of cells expressing PD1 (data not shown). PD1+ CD8+ T cells that failed to produce TNFα and IFNγ were also present to varying degree in all patients (Supplementary Figure 2E, F). Some of these cells could be exhausted myeloma-specific CD8+ T cells, but it is also possible that they were directed towards other antigens. Interestingly, we found fewer PD1+ cells that failed to secrete IFNγ than cells without TNFα production in the bone marrow of the patients. This could be due to the fact that some effector functions more readily inhibited by PDL1-PD1 ligation than others, e.g. TNFα secretion may be easier to inhibit than IFNγ secretion [18].

### PD1+ CD8+ memory and effector cells detected in the bone marrow of myeloma patients

Since terminal effector T cells and memory T cells reside in the bone marrow [14], we set out to determine the proportion of effector and memory cells within the PD1+CD8+ populations. Terminal T effector cells, also called Temra (CD45RA+, CD45RO-, CCR7-) and memory T cells which is divided into T effector memory (Tem) (CD45RA-, CD45RO+, CCR7-) and T central memory (Tcm) (CD45RA-, CD45RO+, CCR7+), was determined (Supplementary Figure 3A). Both T effector and memory cells were found in the in the bone marrow of patients (Figure 2D). Most of the memory cells were effector memory cells rather than central memory cells (Figure 2E).

However, in this group of patients (Supplementary Table 1) and controls tested (Figure 2F), we found that memory cells contributed approximately half of the PD1+ CD8+ T cells in healthy people, with little variation between subjects (median 46.94% and coefficient of variation 17.54%). There was substantially more variation among the myeloma patients (median 22.32% and coefficient of variation 75.15%). Some patients had (Figure 2F), similar to controls, a large proportion of PD1+ memory cells, whilst others had very few or none. This variability could not be attributed to tumor load or other clinical data (data not shown). Effector T cells were also present within the PD1+ CD8+ T cell population in both healthy controls and patients and there were more PD1+ terminal effectors in the patients than in the controls (Figure 2F, Supplementary Figure 3B). In contrast to the memory cells, the proportions varied in both in the patients (median 50.40, coefficient of variation 48.10), and in the control group (median 29.60 coefficient of variation 52.15) (Figure 2F, Supplementary Figure 3B). Many of the memory and effector cells secreted TNFα, however, there were also PD1+ CD8+ effectors and memory cells present in patient bone marrow that failed to secrete TNFα (Supplementary Figure 3C). These cells could be exhausted/dysfunctional cells, potentially able to respond to checkpoint inhibitors.

### Tumor load above 10% PC is associated with increased number of PD1+CD8+EOMES^high^Tbet^low^ cells

**Figure 3:**
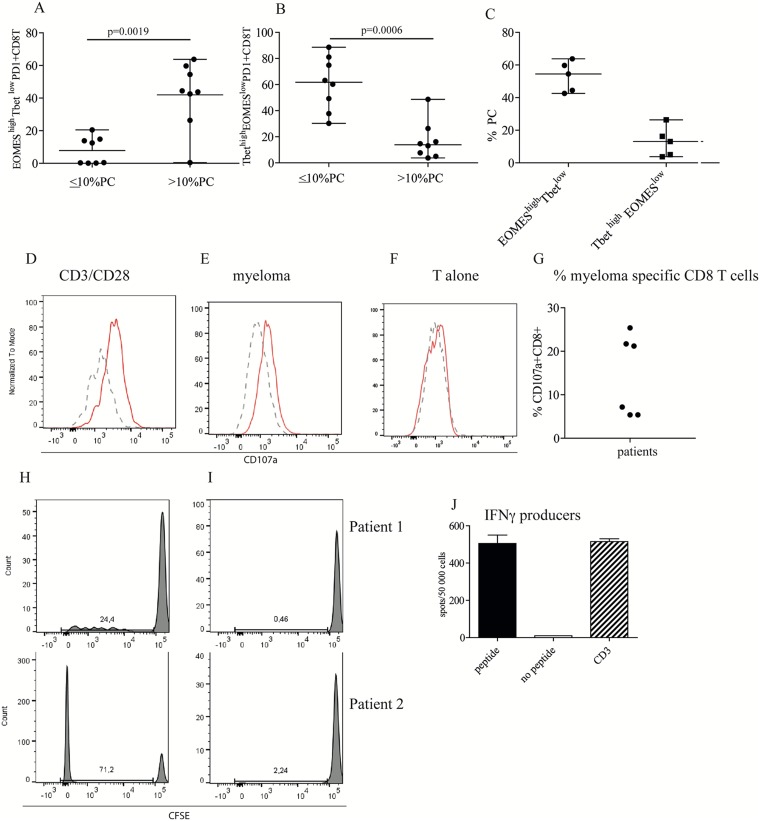
High tumor load is associated with increased number of CD8+EOMES^high^ T bet^low^ cells **(A, B, C)** Frequency of EOMES^high^ Tbet^low^ of PD1+CD8+ T cells. Bone marrow cells were surface stained with anti CD3, CD4, CD8 and PD1 followed by intranuclear staining with anti-Tbet and anti-EOMES, or isotype controls (Supplementary Figure 4A). Gates were set on isotype controls. Figure shows % EOMES^high^Tbet^low^ (A) and Tbet^high^EOMES^low^ (B) of PD1+CD8+ T cells in patients with low plasma cell percentage (≤ 10%) or high plasma cell percentage (>10 % PC). (C) % EOMES^high^Tbet^low^ and Tbet^high^ EOMES^low^of PD1 expressing CD8 T cells in patients with high tumor load of 40% and above. **(D-G)** Cytotoxic activity against autologous myeloma cells. Purified CD8+ T cells from the bone marrow of patients were co-cultured with anti CD3/CD28 beads (D) or purified, autologous myeloma cells (E) or the T cells alone (F). For the last 4 hours of the culture period, anti CD8, PD1 and CD107a or isotype controls were added as described in the Materials and Method section. Figures show the staining of isotype control (dashed lines) and CD107a (solid lines) on one representative sample of three positive (Supplementary Figure 4C). (G) Pooled data for cytotoxic activity (% CD107a expression) of CD8+ T cells co-cultured with autologous myeloma cells. **(H, I)** Proliferation of CD8+PD1+ T cells in response to autologous APCs. CFSE labeled CD3+PD1+ cells from the bone marrow of two myeloma patients (1,2) were cultured with (H) or without (I) autologous, adherent cells in the presence of recombinant IL2 as described. **(J)** PD1+ EBV specific CD8+ T cells in the bone marrow of myeloma patients. PD1+ cells enriched from the bone marrow was cultured with autologous, adherent cells as described in Materials and Methods in the presence of EBV specific peptides. After 10 days of culture the cells were seeded onto IFNγ ELIspot strips together with differentiated DC and EBV peptides (filled bar), without peptides (open bar) or with anti CD3 antibody (striped bar). Spots were developed after 24 hrs. Figure shows representative data from one of the 6 EBV positive patients tested. Data from all the 6 patients are shown in Table 1.

Since PD1 expression alone is not always a measure of T cell exhaustion, we decided to estimate how many exhausted CD8+ T cells there were within the PD1+CD8+ T population by determining the expression of the transcription factors Tbet and EOMES. Tbet and EOMES are involved in regulation of T cell function and generation of T cell memory [19]. A strong TCR signal will promote Tbet activation and generation of short-lived effector cells whilst with more moderate activation, activation via EOMES is preferred and long lived memory cells generated [19]. In chronic infection (and probably cancer), when the cells are exposed to persistent antigenic stimulation, CD8+ T cells will not develop into memory cells, but become exhausted/dysfunctional with upregulation of inhibitory molecules. In this situation, Tbet will improve the functional durability of the CD8+ T cells whilst EOMES will promote dysfunction [20, 21]. We therefore typed a group of myeloma patients for EOMES and Tbet expression, (Supplementary Figure 4A, patient data in Supplementary Table 2), and found that the patients with tumor load of > 10% bone marrow PC had more ‘exhausted’ EOMES^high^Tbet^low^ cells amongst their PD1+ CD8+ T cells than the patients with tumor load ≤10% PC (Figure 3A). This was even more evident in patients with very high tumor load of ≥ 40% PC (Figure 3C). In contrast, the patients with ≤ 10% PC had more Tbet^high^EOMES^low^ within their PD1+CD8+ T cell population than the patients with tumor load of > 10% PC (Figure 3B). All but one patient had PD1+ CD8+ T cells with intermediate levels of PD1, a phenotype that is believed to be early exhausted cells and responsive to PD1+ inhibition (Supplementary Figure 4B) [9]. This suggests that PD1+ exhausted (and possibly myeloma specific) CD8+ T cells may be present in or near the tumor site in myeloma patients.

### The PD1+ CD8+ T cells did not kill autologous myeloma cells *in vitro* and virus specific CD8+ T cells were found in the in the PD1+ population

We established that PD1+CD8+ T effector and memory cells as well as dysfunctional/exhausted cells were present in the bone marrow of myeloma patients, but we do not know what they recognize. In an attempt to determine the specificity of these cells, i.e. whether any of them were directed against autologous myeloma antigens, we tested for expression of CD107a on the CD8+ T cell surface after co-culture of myeloma cells and autologous CD8+ T cells. Detection of CD107a (LAMP1) expression on the surface of CTL by Flow cytometry is an established method of determining cytotoxic activity [22] as CD107a is present together with Granzyme B in the cytotoxic granules and exposed on the cell surface when the cells degranulate. Due to limited availability of material, we tested only six myeloma patients for the expression of CD107a after co-culture with autologous myeloma cells. Figure 3D shows that T cells stimulated with anti CD3/CD28 beads upregulated CD107a on many CD8+ T cells. CTL activity above background levels was also observed after co-culture of T cells and myeloma cells, but not when the T cells were cultured alone (Figure 3E, F). Cytotoxic activity against autologous myeloma cells was found in half of the patients tested (Figure 3G, Supplementary Figure 4C), again this could not be attributed to any clinical parameters (data not shown). The cytotoxic activity was not generated by the PD1+ T cells, but detected in the PD1 negative fraction (Supplementary Figure 4D). Adding anti-PD1 antibody to the co-culture of autologous T cells and myeloma cells did not increase the percentage CD107a+ positive cells or the amount of CD107a expressed (Supplementary Figure 4E). Although we found no evidence of degranulation of PD1+ T cells when co-cultured with autologous myeloma cells, the PD1+ CD8+ T cells proliferated when co-cultured with adherent cells from autologous bone marrow (Figure 3H), but not when cultured on their own (Figure 3I), suggesting that the PD1+ CD8+ T cells may respond to autologous APC (antigen presenting cells). Finally, we investigated whether there were PD1+ CD8+ T cells against other antigens than tumor antigens present in the bone marrow of myeloma patients. Approximately half of the Caucasian European population is seropositive against EBV virus. We therefore stimulated purified CD8+PD1+ T cells with autologous APC and a mixture of EBV peptides restricted to the most common Caucasian European HLA haplotypes and tested for IFNγ producers in an ELIspot assay. Anti EBV activity was found in PD1+ CD8+ cells in bone marrow of patients (Figure 3J and Table 1).

**Table 1:** Number of INFγ producing PD1+CD8 T cells per 50 000 from the bone marrow of myeloma patients

Patient	CD3 (mean)	EBV Peptide (mean)	No peptide (mean)
3125	26125	40	1
3126	109	99	0
3147	515	505	10
3249	279	250	100
3259	TF	158	57
3260	109	99	1

## DISCUSSION

There is limited knowledge on how checkpoint therapy works on the cellular level in myeloma patients. When characterizing TILs in myeloma patients we have to bear in mind that the bone marrow is an immunological organ in addition to a tumor site. The usual conception is that checkpoint therapy activates PD1+ exhausted T cells [12], however, recent studies indicate that PD1+ memory cells may also be targets [23]. Several of the PD1+ CD8+ T cells in the bone marrow recognize antigens other than myeloma antigens.

With that in mind, it is not surprising to find that healthy controls and patients have similar proportions of PD1+ cells and expression levels of PD1 on their CD8+ T cells. Likewise, if many of the PD1+ CD8+ T cells were specific to other antigens, one would not expect correlation between tumor load and percent PD1+ cells or correlations between tumor load and level of PD1 expression. Indeed, we found PD1+CD8+ T cells specific to EBV in the bone marrow of myeloma patients. These are most likely memory cells generated in previous infections as memory cells are located in the bone marrow and in addition, none of the patients tested showed any signs of an acute EBV infection. It would be intriguing to find out how these cells behave during immune therapy.

Estimating proportion of CD8+ T cells with high levels of PD1 and other inhibitory molecules was previously used to determine the number of exhausted (and recoverable) CD8+ T cells. However, CD8+ T cells with very high levels of PD1 were found to be ‘over exhausted’ and beyond recovery by checkpoint therapy. It was now reported that exhausted, EOMES^high^, Tbet^low^ with lower levels of PD1 are the cells that respond to check point therapy [24]. Thus, typing for EOMES and T bet as markers of responsive exhausted cells may be better than determining expression of PD1 and other inhibitory molecules. Indeed, we found PD1ntermediate CD8+ EOMES^high^ Tbet^low^ cells in patients with high tumor load, suggesting that at least some of the PD1+ cells were bona fide exhausted cells and potentially responsive to PD1+ inhibition. Indeed, our findings are supported by the data by Chung et. al. who found PD1+ exhausted/senescent CD8 T cells in the blood of patients that relapsed after ASCT that could regain alloreactivity *in vitro* after adding anti PD1 antibody [25].

It was previously reported that relapsed patients had more PD1+CD8+ T cells and higher levels of PD1 on their cells [3, 7]. We did not find the same in our study, as our patients with high expression levels of PD1 were a mixture of untreated and relapsed patients, and there was no significant difference in either percent or level of PD1 expressed on CD8 T cells from newly diagnosed and relapsed patients (data not shown). PD1+CD8+ T cells with reduced ability to respond to anti CD3 stimulation was detected in the bone marrow of myeloma patients in a recent publication [26]. Whether these were exhausted, memory or effector cells was not shown in that study.

Not surprisingly, we found both PD1+ effector cells and memory cells in the bone marrow of patients. Not all of them may be tumor specific, but the fact that there were a lot more terminal effector cells in the bone marrow of patients than in healthy controls suggested that CD8+ T cells were activated by the tumor. This could be due to either tumor specific or bystander stimulations. Solid tumors with extensive T cell infiltration respond better to checkpoint therapy than tumors without T cell infiltration [27]. The presence of memory cells is particularly important as cancers with memory cell infiltration have a better prognosis [28]. It was recently proposed that CD45RO^+^CCR7^-^ tissue resident memory cells (Trm) were the most likely target of checkpoint inhibitors [23]. We found PD1+CD45RO^+^CCR7^-^ memory cells in the bone marrow of patients. We have called these cells effector memory, but they have the same markers as Trm and could very well be Trms. The fact that memory cells are present in the bone marrow could therefore indicate that the myeloma patients would respond to checkpoint therapy. There were lower proportions of memory cells in patient bone marrow than controls. This was mainly due to significantly lower numbers of PD1+Tcm. It would be interesting to know whether this also contribute to the increased susceptibility to infections found in some patients and/or whether these cells could be responsible for the adverse effects seen in some patients treated with check point therapy.

We attempted to determine, in a small number of patients where enough fresh material was available, whether the PD1+CD8+ T cells were specific to the tumor by adding anti PD1 antibody to the cultures, but the PD1+ cells still failed to degranulate in the co-cultures. PDL1 was expressed on these myeloma cells (data not shown) and we have also reported previously that myeloma cells from patients express PDL1 [5], thus lack of PDL1 expression was not the reason for the failure to degranulate.

However, this could suggest that the PD1+ T cells were either specific to other antigens or specific to myeloma antigens, but functionally exhausted. The fact that they all responded when treated with strong stimuli such as CD3/CD28, indicated that the cells were not terminally exhausted and still responsive. From our data it was difficult to determine whether the PD1+ cells were myeloma specific. This is a technical issue and probably a reason for the limited numbers of reports of autologous, tumor-specific CTL activity in myeloma [7]. IFNγ production and T cell proliferation induced by autologous myeloma cells is well documented [4] [29], and we found that PD1+ autologous CD8+ T cells proliferated in response to APC from the bone marrow.

Monotherapy with checkpoint inhibitors does not work well for all cancers and all patients, but combinations of therapies improve the response [30]. In myeloma anti-PD1 combined with immunomodulatory drugs (IMiDs) gave positive responses in myeloma patients [31]. However, unwanted outcomes in some of these combination trials with IMiDs and the anti-PD1 inhibitor pembrolizumab (Keynote-183 and Keynote-185 clinical studies) have also been reported. Monotherapy with checkpoint antibodies have not been explored fully as only 27 patients were included in the trial [1] and perhaps stratifying patients to include patients with high PDL1 expression may be beneficial as was seen in patients with NSCLC [32]. Importantly, the sequence and timing of therapies to allow for specific priming of tumor cells should be explored. It is at present not clear how anti-PD1-PDL1 works *in vivo*, in particular in combination with other immune regulatory drugs. The bone marrow is the tumor site in multiple myeloma, but also an organ where immune activation takes place where naïve and antigen specific CD8+ T cells, which recognize a variety of different antigens are found [33]. Combining checkpoint antibodies with IMiDs may lead to nonspecific stimulation of T cells that could be potentially harmful.

In conclusion, our data showing the presence of PD1intermediate EOMES^high^Tbet^low^, as well as PD1+ memory cells in patients with tumor load above 10% bone marrow plasma cells could give an indication that checkpoint inhibition would be beneficial if given at the correct time when there are enough tumor responsive CD8+ T cells around. However, better dissection of immune responses in clinical studies of patients treated with checkpoint inhibitors and IMiDs is needed. It is also important to understand how T cells with other specificities behave and influence anti-tumor responses during checkpoint inhibition. It would be interesting to know whether checkpoint inhibitors or checkpoint inhibitors in combination with IMiDs could influence the activity of memory cells to other antigens, including autoantigens.

## PATIENTS AND METHODS

### Bone marrow samples

Bone marrow cells were collected in sodium heparin (Wockhardt) from the iliac crest from healthy controls and patients suffering from multiple myeloma. The patients were registered in the Norwegian Myeloma Biobank and enrolled in the study. The study was approved by the Regional Ethics Committee (REK 2016-81). The donors were classified as healthy, MGUS or multiple myeloma according to the International Myeloma Working Group (IMWG) criteria [34]. Patient's data is shown in Supplementary Table 1 where the patients are grouped according to figures. Samples were obtained after written consent. Bone marrow PC percentage was determined in May Grunwald Giemsa stained smears as part of standard diagnostic procedures. Bone marrow aspirate from healthy donors (mean age 57.33, 8 females, 4 males) were collected the same way as patients (mean age 63.71, 35 females, 67 males).

### Antibodies and reagents

Anti-human CD4FITC (eBioscience 11-0049)), CD3PerCpCy5.5 (eBioscience 45-0037), CD8PE (eBioscience 12-0088), CD45ROFITC, CD45RAPE, TNFα eFlour450 (eBioscience 48-7349), IFNγAPC (eBioscience 17-7319), TbetPE (eBioscences 12-5825-80), eomesPE-eFlour610 (eBioscience 61-4877) and appropriate isotype control antibodies as well as human Fc Receptor binding inhibitor (eBioscience 14-9161), Protein transport inhibitor (Brefeldin A and Monensin (eBioscence 00-4980)), PMA and Ionomycin (T cell stimulation cocktail (eBioscience 00-4970 )), Intracellular fixation and permeabilization kit (eBioscience 88-8824) FOXP3/transcription factor staining kit (eBioscience 00-5523). FACS lysing buffer was obtained from BD Biosciences (BD 349202). CD279 PECy7 (BioLegend 329917), CD8AF700 (BioLegend 301028), CCR7APC (BioLegend 353214), GranzymeBAlexaFlour647 (BioLegend 515405), CD107eFlour450 (eBioscience 48-1079) and appropriate isotype controls. CD3/CD28 activator beads (Gibco 11131D), EBV peptides; HLA A2.1 (GLCTLVAML), HLA A2.2 (CLGGLLTMV), HLA A2.3 (LLDFVRFMGV), HLA B7.1 (RPPIFIRRL), HLA B7.2 (VPAPAGPIV), HLA B8.1 (RAKFKQLL), HLA B8.2 (QAKWRLQTL), HLA B8.3 (FLRGRAYGL). Peptides were obtained from Invitrogen.

### Flow cytometry

Crude bone marrow cells were stained with a cocktail of antibodies for 30 min on ice after 20 min incubation with human Fc Receptor binding inhibitor. RBCs were lysed and cells fixed after staining. Flow cytometry was performed using LSR II (BD Biosciences) with FACS Diva software (BD Biosciences). Samples were analysed with FlowJo 10.4 (TreeStar). Gates were set on live cells with forward and side scatter and doublets were gated out.

### Intracellular cytokine staining

For cytokine detection, bone marrow mononuclear cells (BMMC) were collected from bone marrow aspirates after density centrifugation with Lymphoprep (Axis Shield 111544). 2 × 10^6^ BMMC were then cultured in 500 μl RPMI (10% human serum)/ well in a 24 well plate (COSTAR) at 37°C for 2 hr together with cell stimulation cocktail before adding protein transport cocktail for another 2 hr. Intracellular staining was performed after surface staining followed by PFA fixation and permeabilization using the manufacturer's protocol (eBioscience 88-8824).

### Intranuclear staining

RBCs in bone marrow aspirates were lysed with hypotonic ammonium chloride (eBioscience 00-4300) for 5 minutes at 37°C. The cells were surface stained with anti CD4FITC, CD8PE, CD3PerCpCy5.5 and PD1PECy7 as described. The cells were then fixed, permeabilized and stained with anti TbetPE and eomes PEeFlour610 using the FOXP3/transcription factor staining kit following the manufacturer's protocol (eBioscience 00-5523).

### Purified CD138+ plasma cells

CD138+ plasma cells were obtained from the mononuclear cell fraction of bone marrow aspirate of myeloma patients as previously described [35]. The plasma cells were > 95% pure.

### Cytotoxic T cell assay

CD8+ T cells were obtained from the unbound section of bone marrow aspirates after positive selection using anti CD138 magnetic beads (Stem Cell Technologieies 18387) as described above. The CD8+ T cells were enriched by positive selection with magnetic beads (Miltenyi Biotech 130-045-201). 2×10^5^ T cells and 2×10^5^ autologous CD138+ plasma cells were co-cultured for 4–7 days in 96 well round bottom plates in RPM1 with 10% human serum. Labelled anti-PD1, CD8, CD107a or isotype control was then added to the culture and incubated at 37°C in the presence of Protein Transport Inhibitor for 4 hr. The cells were then fixed with 2% PFA and analyzed with LSRII as described. CD3/CD28 activator beads were used at (1:1) to stimulate the T cells. Anti PD1 (BioLegend 329911) or isotype control (BioLegend 401404) were used at 10μg/mL in blocking experiments.

### T cell stimulation and proliferation

2 × 10^6^ cells from the unbound section of bone marrow aspirates obtained after positive selection with anti CD138 magnetic beads [35] was adhered to wells of 96 wells of flat bottomed tissue culture plates for 1 hr at 37°C. CD3+T cells were enriched to > 50% purity from the non-adherent population by negative selection (Invitrogen 11344D). CD3+PD1+ T cells were further enriched using biotinylated anti-PD1 (BioLegend 329934) and streptavidin microbeads (Miltenyi Biotech 130-048-102). 1×10^5^ PD1+ T cells were incubated with CFSE (Invitrogen C34554) following the manufacturer's instructions. The cells were then added to the autologous, adherent cells and incubated in RPMI (10% human serum), 20 U/mL rIL2 (R&D systems 202-IL-010) for 6 days. The cells were then harvested, stained with anti CD3 and CD8 antibodies and analyzed on LSRII.

### ELIspot analysis of EBV specific CD8+ T cells

PD1+ cells and adherent cells were obtained from the unbound section of bone marrow aspirates after positive selection using anti CD138 coated beads as described. APCs were isolated by adherence to 24 well tissue culture plates for 1 hr at 37°C. PD1+ cells were enriched to > 80% purity from the non-adherent fraction using anti-PD1 biotinylated antibody (BioLegend 329934) and streptavidin beads (Miltenyi Biotec 130-048-102). 5-10 × 10^5^ PD1+ cells were added to the adherent cells in RPMI 10% human serum with a mixture of each 2 μg/mL EBV peptides and cultured for 10 days at 37°C. 20 U/ml recombinant IL2 (R&D systems 202-IL-010) was added on days 3 and 7. In separate wells dendritic cells (DC) were differentiated from adherent cells in RPMI 10% human serum and 20ng/mL GM-CSF (R&D systems 215-GM-010) and 50ng/mL IL4 (PeproTec 200-04). The DCs were dislodged with Accutase (Sigma A6964) and harvested after day 10 of culture. 5-10 × 10^4^ T cells/well was co-cultured with differentiated DC on anti IFNγ coated ELIspot strips (MABTECH 3420-4AST-2) in the presence or absence of EBV peptides. Anti CD3 antibody was used as control. Spots were developed after 24 hr incubation following the manufacturer's protocol.

### Statistical analysis

Tests were performed using Graph Pad Prism 5 software. Correlations were determined using Spearman's correlation ranks test. Comparisons between groups were done with Mann–Whitney *U* test. Significance was determined as p< 0.05.

Authors: AMS-performing and designing experiments, conceived idea, writing paper, RY-performing experiments, AW, EHR, TSS clinical data and writing paper, AST,CDV collecting clinical data, TS, MB writing paper, AS writing paper, conceived idea.

## SUPPLEMENTARY MATERIALS FIGURES AND TABLES




