# Supplementary material for: PD1 is expressed on exhausted T cells as well as virus specific memory CD8+ T cells in the bone marrow of myeloma patients
Source: Oncotarget. 2018 Aug 10;9(62):32024–35. doi: 10.18632/oncotarget.25882 (PMC6112830; doi:10.18632/oncotarget.25882)
Supplement: Supplementary file 1 [file oncotarget-09-32024-s001.pdf]

PD1 is expressed on exhausted T cells as well as virus specific memory CD8+ T cells in the bone marrow of myeloma patients

SUPPLEMENTARY MATERIALS

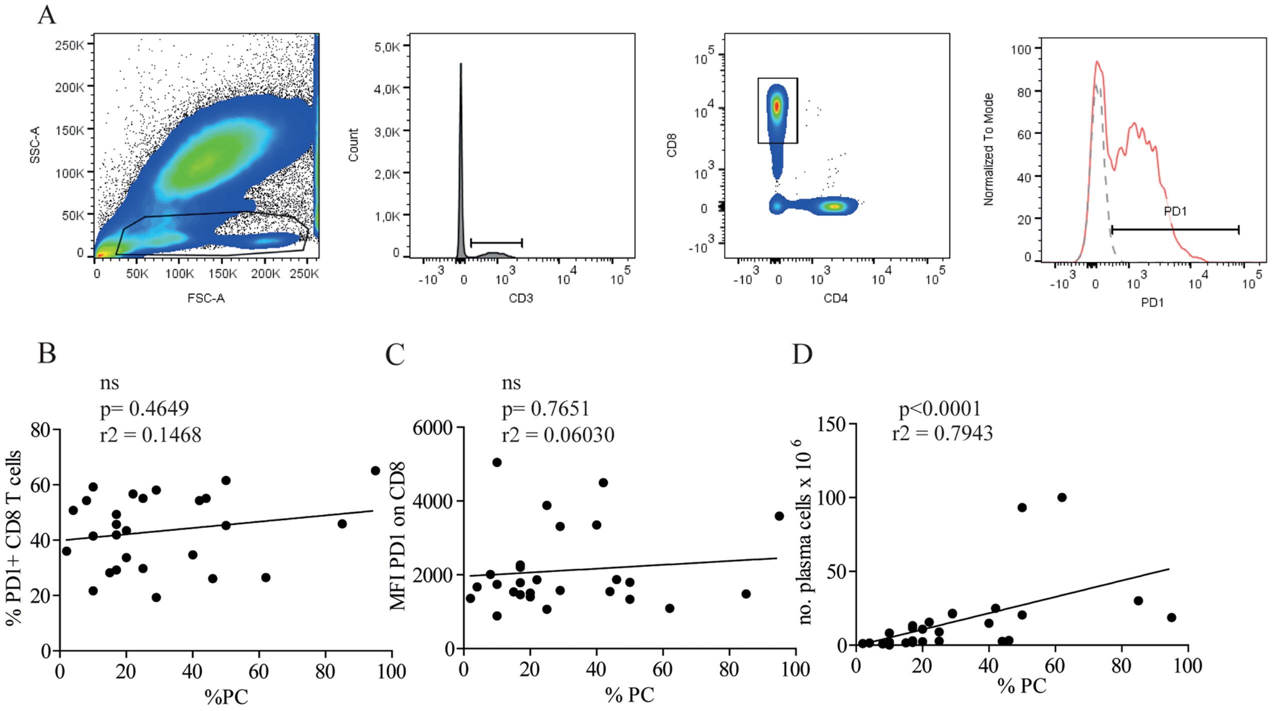

Supplementary Figure 1: (A) Gating strategy of a representative patient sample. Gates were set on FSC and SSC and doublets were excluded. Gates were then set on CD3 and CD8 and figure shows a histogram of PD1+ expression on CD8+ T cells (solid red line). FMO controls were used to determine percent PD1+ cells (dashed grey line). **(B, D)** Correlation of % plasma cells and % PD1+CD8+ T cells (B) and MFI of PD1 on CD8+ T cells **(C)** from patients in Figures 1 A/B. Correlation of number of CD138+ plasma cells obtained from 20 mL bone marrow aspirate and % plasma cells in Giemsa stain from the same patients (D). Each dot represents one patient.

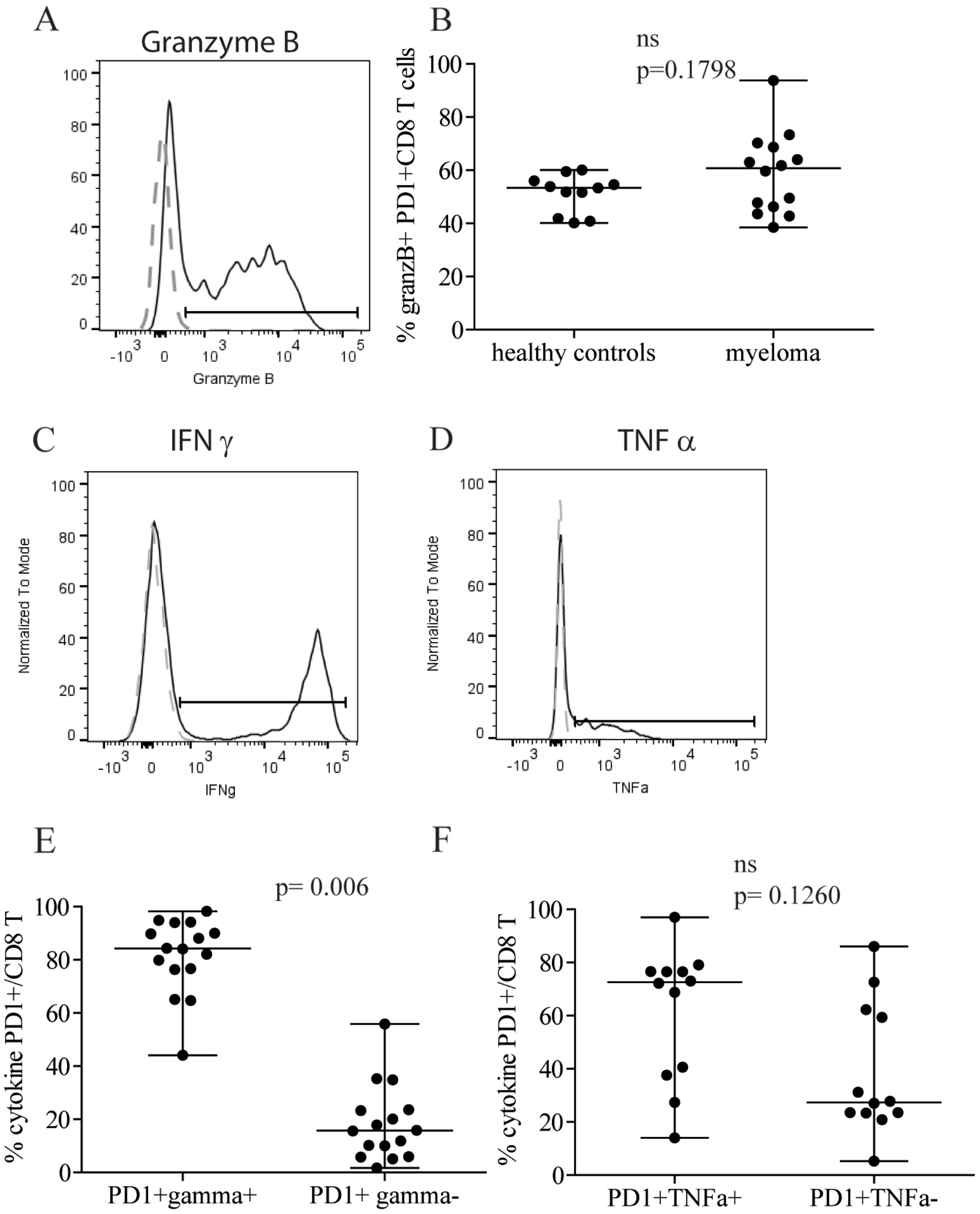

Supplementary Figure 2: **(A)** Histogram of Granzyme B expression (solid line) on gated PD1+ CD8+ T bone marrow cells from a representative patient. Crude bone marrow was surface stained with anti CD3, CD4, CD8 and PD1 followed by intracellular staining with anti Granzyme B or isotype control. Gating strategy is shown in Supplementary Figures 1A and 2A. Gates were set on isotype control (dashed line). **(B)** Distribution of % Granzyme B+ cells within the PD1+CD8+ T cell population in the bone marrow of healthy control (n=11) and myeloma patients (n=14). Figure shows median value and range. **(C, D)** Histogram of IFNγ (C) and TNFα (D) expression (solid line) on gated PD1+ CD8+ T cells in bone marrow from a representative patient. Gates were set on isotype control (dashed line). **(E, F)** Percent cytokine producing and non-producing cells PD1+CD8+ T cell population in the bone marrow of patients. BMMCs were stimulated, stained and gated as described in Figure 2. Anti IFNγ (n=16) (E), TNFα (n=12) (F).

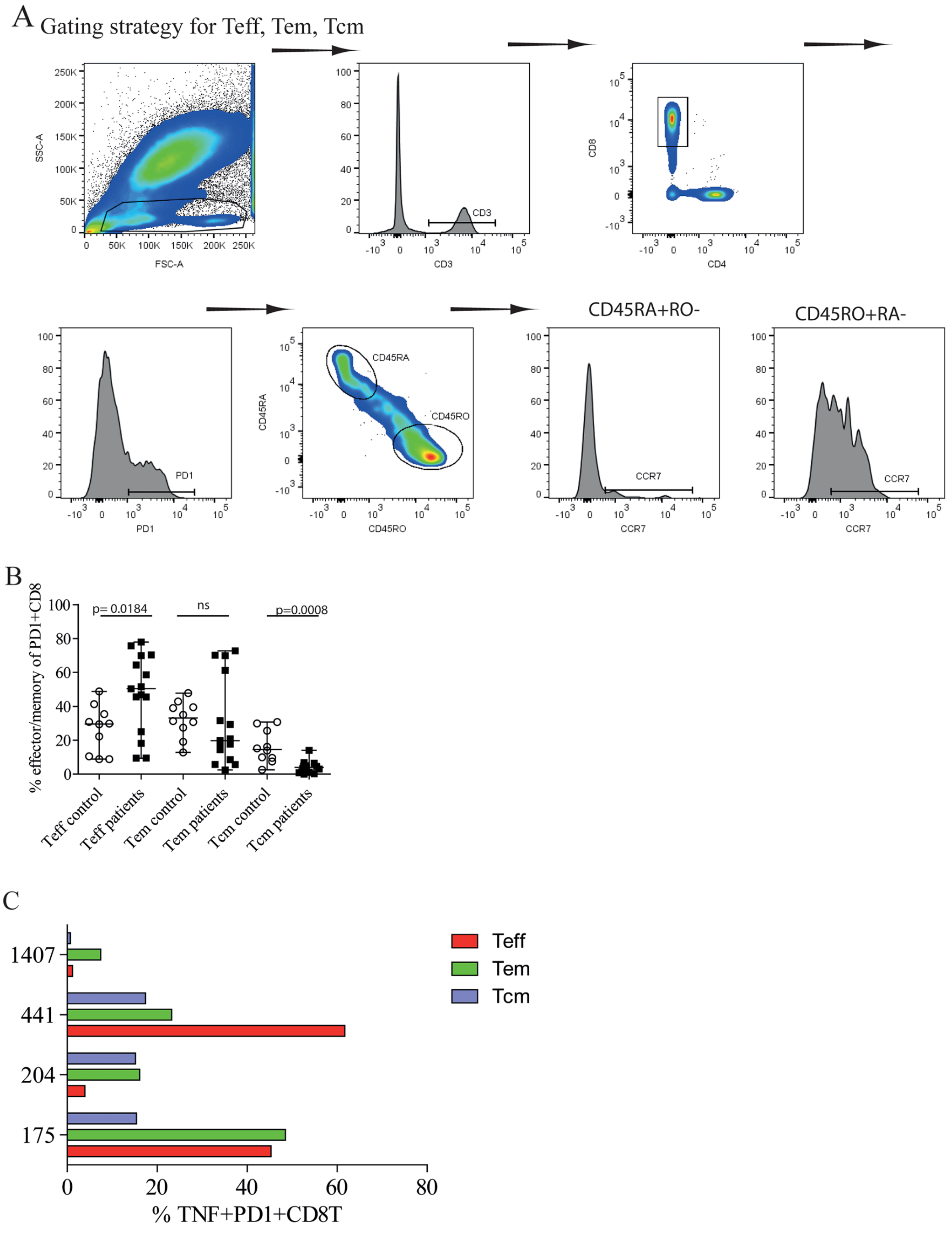

Supplementary Figure 3: (A) Gating strategy for PD1+CD8+ Teff, Tem and Tcm. Crude bone marrow cells from myeloma patients and healthy controls were stained with antibodies to CD3, CD4, CD8, PD1, CD45RA, CD45RO and CCR7 as described. Gates were set on FSC and SSC and doublets were excluded. Gates were then set on CD3 and CD8 followed by PD1. Gates were further set on CD45RA+RO- and CD45RA-RO+ populations and CCR7- and CCR7+ populations were determined within the CD45RA+RO- and CD45RO+RA- populations by Boolean gating in FlowJo. **(B)** Frequency of T effector, Tem and Tcm PD1+CD8+ bone marrow cells in myeloma patients and healthy controls. Figure shows median value and range. **(C)** TNFα secretion in PD1+CD8+ Teff (CD45RA+CD45RO-CCR7-), Tem (CD45RO+CD45RA-CCR7-) and Tcm (CD45RO+CD45RA-CCR7+) cells in the bone marrow of four myeloma patients. BMMC were stimulated described in Materials and Methods. The cells were then surface stained with anti CD3, CD4, CD8, PD1, CD45RO, CD45RA and CCR7 followed by intracellular staining with anti TNFα or isotype control.

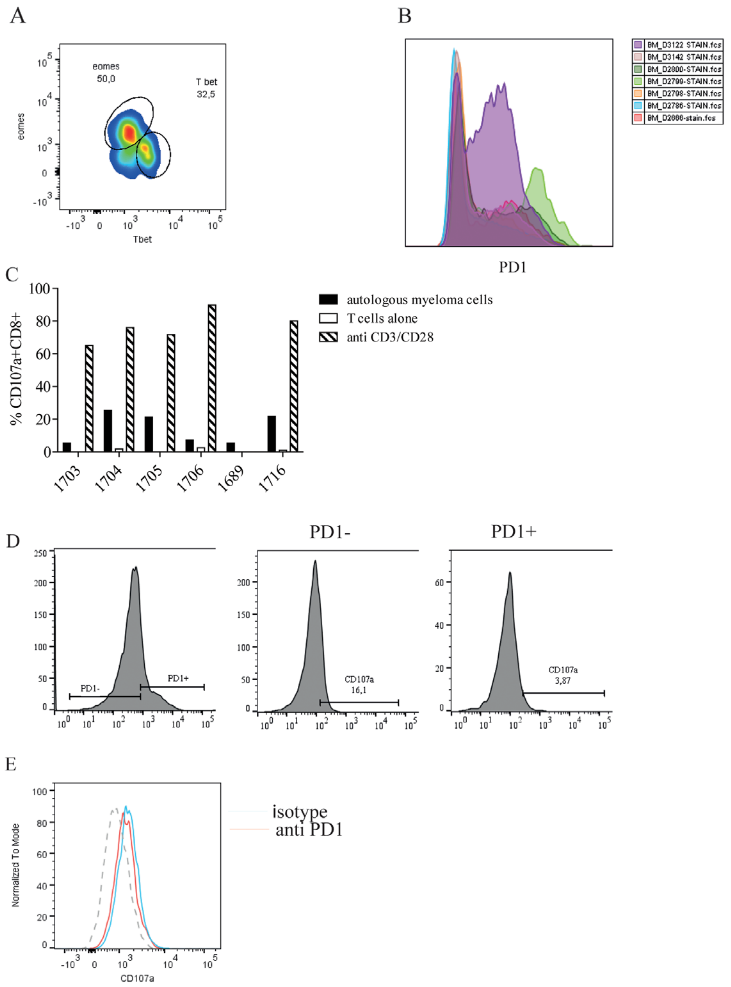

Supplementary Figure 4: **(A)** Plot of expression of EOMES and Tbet on gated PD1+CD8+ T cells. Bone marrow cells were surface stained with antibodies against CD3, CD4, CD8 and PD1, fixed and stained intra-nuclear with anti Tbet and EOMES as described in Materials and Methods. Gated $EOMES^{high}Tbet^{low}$ and $Tbet^{high}EOMES^{low}$ from a representative patient is shown. **(B)** Histogram of PD1 expression on gated CD8+ T cells from patients with >10% PC in the bone marrow. **(C)** Expression of CD107a on bone marrow CD8+ T cells from the 6 patients tested. Cells were cultured with autologous myeloma cells (filled bars), alone (open bars) or with anti CD3/CD28 beads (striped bars) as described and stained with anti CD8, PD1 and CD107a. Figure shows % CD107a+CD8+ T cells. Gates were set on isotype control. **(D)** No cytotoxic activity (CD107a expression) against autologous myeloma cells in the PD1+ CD8+ T cell populations. PD1 staining of CD8+ T cells from a representative patient after co-culture with autologous myeloma cells and purified CD8+ T cells. Histograms of CD107a expression on PD1+ and PD1- CD8+ T cells. Gates were set on isotype control. **(E)** Presence of PD1 antibody does not increase the expression of CD107a on the CD8+ T cells. CD8+ T cells were cultured with autologous myeloma cells and stained with anti CD107a as described in the presence of anti-PD1 (red line) or isotype control (blue line). (Striped line is conjugated isotype staining control).

Supplementary Table 1: Patient data

See Supplementary File 1

Supplementary Table 2: Table of % PC and % $EOMES^{high}Tbet^{low}$ and % $Tbet^{high}$ $EOMES^{low}$

Patient	% PC	$\%eomes^{hi}Tbet^{low}$	$\%Tbet^{hi}eomes^{low}$
2786	42	54.5	13.1
562	10	0.1	88.6
2693	7	0.3	81.1
2666	50	42.6	26.4
2752	10	0.2	63.2
2838	20	0.4	48.7
2798	17	26.4	14.7
2799	32	43.8	7.7
2800	40	44.4	16.2
3020	8	20.5	30.3
3022	8	14.8	37.8
3121	10	0.1	74.9
1256	5	12.5	49.1
828	10	13.8	60.3
3142	60	63.8	3.8
3122	42	59.7	5.0
